# 晚期*EGFR*突变型非小细胞肺癌患者接受吉非替尼或厄洛替尼治疗的成本效益分析

**DOI:** 10.3779/j.issn.1009-3419.2013.04.06

**Published:** 2013-04-20

**Authors:** 宇翔 马, 岩 黄, 洪云 赵, 俊玲 刘, 丽昆 陈, 海鹰 吴, 宁宁 周

**Affiliations:** 510060 广州，中山大学附属肿瘤医院内科 Department of Medical Oncology, Sun Yat-sen University Cancer Center, Guangzhou 510060, China

**Keywords:** 吉非替尼, 厄洛替尼, 肺肿瘤, 成本效益分析, efitinib, Erlotinib, Lung neoplasms, Cost-effectiveness analysis

## Abstract

**背景与目的:**

非小细胞肺癌(non-small cell lung cancer, NSCLC)靶向治疗越来越受到关注, 吉非替尼和厄洛替尼均被推荐用于存在表皮生长因子受体酪氨酸激酶(epidermal growth factor receptor, EGFR)基因突变的晚期NSCLC的一线治疗。本研究旨在分析比较吉非替尼和厄洛替尼在晚期NSCLC的疗效和生存获益, 以及治疗成本效益。

**方法:**

回顾性分析广州医保内的66例*EGFR*突变型的NSCLC患者。观察疗效和记录不良反应, 定期随访生存预后, 并追踪治疗费用。

**结果:**

总共66例可评估患者, 中位无进展生存期(progression-free survival, PFS)为15.0个月。其中吉非替尼49例, 厄洛替尼17例, PFS分别为17.5个月和13.0个月(*P*=0.459)。皮疹发生率吉非替尼组为62.3% (31/49), 厄洛替尼组为94.1%(16/17)。成本-效益比率(cost-effectiveness ratio, CER)吉非替尼组为3, 027元/月, 厄洛替尼组为6, 800元/月, 增量成本-效益比率(incremental cost-effectiveness ratio, ICEA)厄洛替尼为吉非替尼的2.25倍。

**结论:**

*EGFR*突变的晚期NSCLC患者治疗, 吉非替尼和厄洛替尼有相似的疗效和生存获益, 前者不良反应可能较为轻微。广州医保下, 吉非替尼成本-效益比率稍优。

## Abstract (en)

**Background and objective:**

Targeted therapy in non-small cell lung cancer (NSCLC) had become a research hotspot.Both of gefitinib and erlotinib had already been recommended as first line treatment in epidermal growth factor receptor (EGFR) mutant advanced NSCLC patients.The study aimed to compare the effectiveness and prognosis of advanced NSCLC with gefitinib or erlotinib, as well as the cost-effectiveness ratio of the two drugs.

**Methods:**

Data of 66 *EGFR* mutant NSCLC patients who were included in Guangzhou medical insurance were analyzed.The efficacy and adverse reactions were evaluated.All the patients were followed-up regularly and the cost of the treatment was recorded.

**Results:**

The median progression free survival (PFS) of all patients was 15.0 months.49 patients received gefintib and 17 patients had erlotinib.The PFS for the two groups of patients was 17.5 month and 13 months, respectively (*P*=0.459).31 (62.3%) patients had rash in gefitinib group, 16 (94.1%) in erlotinib group.Cost-effectiveness ratio (CER) in gefitinib group was 3, 027 RMB per month, while 6, 800 RMB in erlotinib group.The incremental cost-effectiveness ratio (ICEA) of erlotinib was 2.25 times of gefitinib.

**Conclusion:**

For *EGFR* mutant advanced NSCLC patients, equal efficacy and survival benefit were observed in patients with gefitinib and erlotinib.The adverse reaction was milder in gefitinib group than that of erlotinib group.And with Guangzhou medical insurance, gefitinib had a superior cost-effectiveness ratio.

肺癌现已成为最致命的恶性肿瘤, 且发病率和病死率一直呈明显的上升趋势^[1]^。在我国, 肺癌也是发病率及病死率最高的恶性肿瘤^[2]^。非小细胞肺癌(non-small cell lung cancer, NSCLC)约占所有肺癌的85%, 30%-40%的NSCLC患者在诊断时即为局部晚期或发生远处转移^[3, 4]^, 化疗是这部分人群的主要治疗手段。但是, 标准一线治疗的含铂两药联合化疗方案的无进展生存期(progression free survival, PFS)为4个-6个月, 中位生存期(overall survival, OS)仅为10个-12个月^[5]^, 其疗效已达平台期, 患者在化疗上的获益似乎已经最大化。随着肿瘤分子生物学的发展和基因水平的研究深入, 靶向治疗已成为目前研究和临床应用的热点。

厄洛替尼(商品名:特罗凯, 罗氏)、吉非替尼(商品名:易瑞沙, 阿斯利康)是较早在中国上市的表皮生长因子受体(epidermal growth factor receptor, EGFR)酪氨酸激酶抑制剂(tyrosine kinase inhibitor, TKI)。INFORM、IPASS、OPTIMAL等几项随机对照研究已证实了EGFR-TKI治疗晚期NSCLC的客观缓解率(objective remission rate, ORR)及PFS在*EGFR*突变人群中有明显优势^[6-10]^。由于其在改善晚期*EGFR*突变型^[11]^的NSCLC患者的生活质量及延长PFS上的重要作用, EGFR-TKI已被纳入广州市市属医保报销范围, 针对人群包括具有*EGFR*外显子19和外显子21基因突变(直接测序法)的晚期NSCLC患者, 让更多患者能接受更为有效的治疗。

本研究回顾性分析近年来本院接受吉非替尼或厄洛替尼治疗的广州医保的NSCLC患者的临床资料, 评价两者的疗效及生存获益以及纳入广州医保支付范围后的成本效益分析, 从而为临床治疗NSCLC寻找一种安全有效而且经济的治疗方案。

## 1 资料与方法

### 1.1 临床资料

回顾性研究分析自2008年9月-2012年9月在我院接受过吉非替尼或厄洛替尼治疗的广州医保66例晚期NSCLC患者。其中49例接受吉非替尼治疗, 17例接受厄洛替尼治疗。

### 1.2 纳入条件

① 患者经病理学或细胞学确诊为Ⅲb期、Ⅳ期NSCLC; ②治疗前需行*EGFR*基因检测, 结果为外显子19缺失(E746)或外显子21(L858R)点突变的*EGFR*突变患者可入组, 检测方法是直接测序法; ③骨髓功能良好。排除条件:严重心肺基础疾病; 影响药物吸收的胃肠道疾病; 有慢性湿疹等皮肤病。

### 1.3 治疗方法

接受吉非替尼250 mg/d或厄洛替尼150 mg/d的治疗。每日定期空腹口服药物。持续用药直到病情进展、死亡或者因不良反应不能耐受为止。

### 1.4 临床观察

常规以CT、MR等影像学手段对目标病灶及非目标病灶进行疗效评价, 在治疗前1周-2周以及治疗开始第4周分别进行一次影像学评价, 此后每8周进行一次影像学评价。按照实体瘤的疗效评价标准(Response Evaluation Criteria in Solid Tumours, RECIST 1.1)标准进行疗效评价。按照常见不良反应事件评价标准(Common Terminology Criteria for Adverse Events, CTCAE v4.0)对不良反应进行评价和记录。主要研究终点为PFS, 次要研究终点为疾病控制率(disease control rate, DCR)、ORR以及安全性。自患者接受治疗开始随访, 随访截止时间为2012年12月6日, 截止时间仍然生存的病例列为截尾数据。

### 1.5 经济学评价方法

随访复查时的实验室检查和影像学检查基本相似, 两者主要差异在药品费用以及药物产生相关的不良反应的治疗费用上。由于不同药物患者的例数和服用时间存在差异, 此文计算平均1个疗程(1个月)所需要的药物费用和相关不良反应的治疗费用。按照我院药品的零售价以人民币计算药物费用:以每个患者治疗1疗程(1个月)的药物费用计算。分析模型见图 1。

**1:**
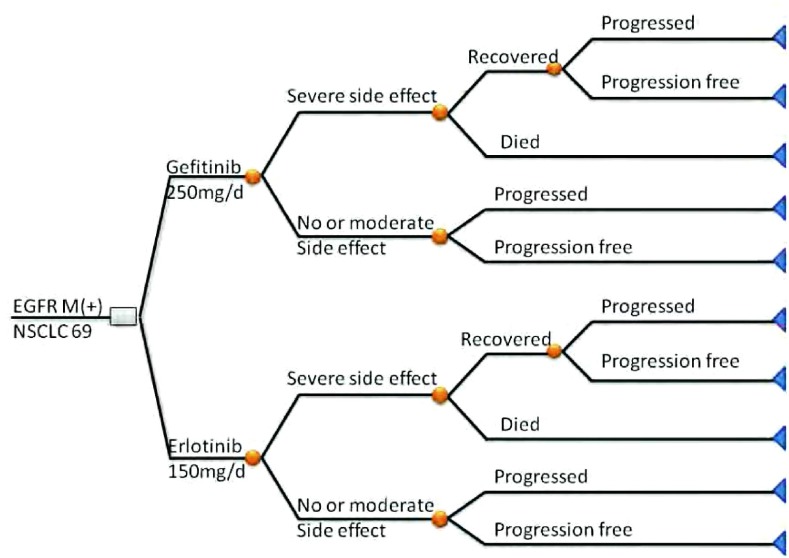
66例入组的*EGFR*突变型NSCLC患者的成本效益分析模型。包括两种治疗方法所使用的药物吉非替尼和厄洛替尼, 以及处理相关治疗副反应。 The cost-effectiveness analysis model of 66 *EGFR* mutant NSCLC patients, including two therapeutic drugs gefitinib and erlotinib, and the treatment of adverse effect.EGFR:epidermal growth factor receptor.NSCLC:non-small cell lung cancer; EGFR:epidermal growth factor receptor.

成本-效益分析:成本如前所诉, 效益标准采用PFS。成本-效益比率(cost-effectiveness ratio, CER)定义为PFS每增加1个月所需要的费用。增量成本-效益比率(incremental cost-effectiveness ratio, ICEA)定义为节省的每1个无进展生存人年(progression-free life-year saved, PF-LYS)的边际成本^[12-14]^。

### 1.6 统计学分析

PFS定义为患者开始口服靶向药物治疗至疾病进展或疾病尚未进展的末次随访时间。OS定义为自患者开始口服靶向药物治疗至患者死亡或末次随访时间。计量资料采用*t*检验; 计数资料采用卡方检验或*Fisher*确切概率法; 生存分析采用*Kaplan-Meier*法, 用*Long-rank*检验分析。所有的统计分析均使用统计软件SPSS 16.0完成, *P* < 0.05认为差异具有统计学意义。

## 2 结果

### 2.1 病例结果特点

66例患者的特征:中位年龄为62岁(39岁-80岁), 其中男性37例(56.1%), 女性29例(43.9%)。ECOG评分均为0分-1分, 两组患者之间年龄、性别、吸烟状态等差异均无统计学意义, 基线状况较为平衡(表 1)。

**1:** 66例非小细胞肺癌患者的一般特征 Characteristics of 66 non-small cell lung cancer (NSCLC) patients

	Tatal	Gefitinib	Erlotinib	*P*
Patients	66 (100%)	49 (74.2%)	17 (25.8%)	
Age (yr)	62 (39-80)	61 (39-80)	64 (42-78)	0.313
Gender				0.087
Male	37 (56.1%)	24 (64.9%)	13 (35.1%)	
Female	29 (43.9%)	25 (86.2%)	4 (13.8%)	
ECOG PS status				
0-1	66 (100%)	49 (74.2%)	17 (25.8%)	
Smoking status				0.249
Former and current	23 (34.8%)	15 (65.2%)	8 (34.8%)	
Never	43 (65.2%)	34 (79.1%)	9 (20.9%)	
Pathological types				0.247
Adenocarcinoma	58 (87.9%)	42 (72.4%)	16 (27.6%)	
Adenosquamous carcinoma	6 (9.1%)	6 (100%)	0 (0)	
Squamous carcinoma	2 (3.0%)	1 (50%)	1 (50%)	
Clinical stages				0.565
Ⅲb	4 (6.1%)	4 (100%)	0 (0)	
Ⅳ	62 (93.9%)	45 (72.6%)	17 (27.4%)	
Mutation type				0.167
19-del (E746)	29 (43.9%)	24 (82.8%)	5 (17.2%)	
21-L858R	36 (54.5%)	24 (66.7%)	12 (33.3%)	
Unknown	1 (1.6%)	1 (100%)	0 (0)	
Treatment lines				0.160
1^st^	23 (34.8%)	14 (68.9%)	9 (31.1%)	
2^nd^	40(60.6%)	33 (82.5%)	7 (17.5%)	
3^rd^ and more	3 (4.6%)	2 (66.7%)	1 (33.3%)	

### 2.2 无进展生存期

从2008年9月开始, 截止到2012年12月6日, 共有18例患者因为肿瘤进展死亡。总体的中位PFS为15.0个月。在所有治疗患者里, 外显子19与外显子21突变的患者的PFS无统计学差异(14.6个月*vs* 15.0个月, *P*=0.97)(图 2A)。吉非替尼组与厄洛替尼组患者的PFS分别为17.5个月*vs* 13.0个月, 差异无统计学意义(*P*=0.459)(图 2B)。对各亚组进行分析, 男性患者吉非替尼组与厄洛替尼组患者的PFS无统计学意义(14.3个月*vs* 13.0个月, *P*=0.909)(图 2C); 女性患者类似(15个月*vs* 5个月, *P*=0.409)(图 2D)。吸烟的患者吉非替尼组与厄洛替尼组患者的PFS无统计学意义(14.8个月*vs* 9.6个月, *P*=0.215)(图 2E); 不吸烟的患者类似(16.4个月*vs* 16.2个月, *P*=0.577)(图 2F)。

**2:**
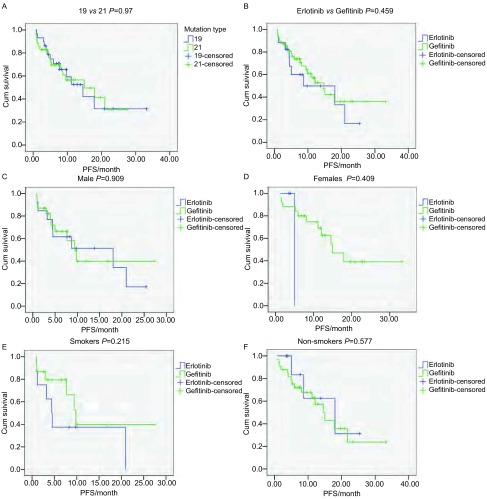
患者的无进展生存及总生存*Kaplan-Meier*生存曲线。A:所有患者中EGFR外显子19缺失对比外显子21点突变的PFS生存曲线; B:口服Gefi组对比Erlo组的PFS生存曲线; C:男性患者中口服Gefi组对比Erlo组的PFS生存曲线; D:女性患者中口服Gefi组对比Erlo组的PFS生存曲线; E:吸烟患者中口服Gefi组对比Erlo组的PFS生存曲线; F:不吸烟患者中口服Gefi组对比Erlo组的PFS生存曲线。 *Kaplan-Meier* curves of PFS and OS.A:PFS comparing between exon 19 mutation and exon 21 mutation in all patients; B:PFS comparing between gefitinib and erlotinib; C:PFS comparing between gefitinib and erlotinib in male patients; D:PFS comparing between gefitinib and erlotinib in female patients; E:PFS comparing between gefitinib and erlotinib in smoking patients; F:PFS comparing between gefitinib and erlotinib in non-smoking patients.PFS:progression free survival.

### 2.3 临床疗效

此项目纳入的66例患者均可评估。吉非替尼组49例, PR 25例(51.0%), SD 20例(40.8%), PD 4例(8.2%); 厄洛替尼组17例, PR 7例(41.2%), SD 8例(47.1%), PD 2例(11.7%)。吉非替尼组DCR为91.1%, 厄洛替尼组为86.7%(*P*= 0.643);吉非替尼组的ORR为51.0%, 厄洛替尼组为41.2%(*P*=0.578)(表 2)。

**2:** 66例患者皮疹与疗效的关系 The relationship between rash and effect of 66 patients

Outcome	Gefitinib	Erlotinib	*P*
PR	25 (51.0%)	7 (41.2%)	
SD	20 (40.8%)	8 (47.1%)	
PD	4 (8.2%)	2 (11.7%)	
DCR (%)	91.1	86.7	0.643
RR (%)	51.0	41.2	0.578
PR:partical response; SD:stable disease; PD:progressive disease; DCR:disease control rate; RR:response rate.			

### 2.4 总体不良反应

发生率最高的是皮疹, 总共42例(63.6%); 其次是腹泻, 总共18例(27.3%); 然后是肝损害(AST或ALT升高), 总共15例(22.7%)。3度以上不良反应的患者都接受过停药以及相应对症处理恢复良好, 并未影响到疗效, 无副作用相关死亡事件。

皮疹的患者PR率为38.3%, SD率为55.3%, 无皮疹的患者PR率为52.6%, SD率为31.6%, 有无皮疹疗效差异无统计学意义(*P*=0.17)(表 3)。

**3:** 66例患者的治疗反应 The treatment response of 66 patients

Outcome	Rash (%)	No Rash (%)	*P*
PR	18 (38.3%)	10 (52.6%)	
SD	26 (55.3%)	6 (31.6%)	
PD	3 (6.4%)	3 (15.8%)	
Total	47 (71.2%)	19 (28.8%)	0.17

吉非替尼组皮疹发生率较低(*P*=0.002);重度(3度或以上)发生率也较低(*P*=0.022)。吉非替尼组腹泻发生率较低(*P*=0.004);重度发生率两者无明显差别(*P*=0.069)。肝损害发生率差别不大(*P*=0.318)(表 4)。本研究是回顾性分析, 病例数较少, 更准确的安全性对比有待前瞻性临床试验得出。

**4:** 66例患者的不良反应 The adverse effects of 66 patients

Group		Rash (%)	Diarrhea (%)	Liver damage (%)
Gefitinib	All	31 (62.3%)	12 (24.5%)	13 (26.5%)
	Grade 3-4	6 (12.2%)	1 (2.0%)	2 (4.0%)
Erlotinib	All	16 (94.1%)	12 (70.6%)	2 (11.7%)
	Grade 3-4	6 (35.3%)	3 (17.6%)	0 (0)
*P*	All	0.002	0.004	0.318
	Grade 3-4	0.022	0.069	
Total		47 (71.2%)	24 (36.4%)	15 (22.7%)

### 2.5 治疗成本-效益分析

两组的PFS无明显差异, 治疗成本的差异则体现在1个疗程(1个月)所需要的药物费用和相关不良反应的治疗费用上。常规情况下, 患者出现症状后使用相关对症处理, 症状都能得到缓解, 其中3例患者因皮疹、3例因腹泻, 以及2例患者因肝损害, 均接受停药并住院治疗, 相关费用也加入了治疗成本中。本研究采用门诊用药系统对这66例患者的EGFR-TKI以及不良反应费用进行分析, 结果如表 5所示。

**5:** 66例患者治疗平均成本对比分析(包括每个周期的靶向治疗药物费用, 治疗药物不良反应的药物以及相应住院费用) The average cost analysis of 66 patients (including the cost of drugs, the treatment of adverse effects, and cost of hospitalization)

Outcome/1 cycle	Gefitinib (RMB)	Erlotinib (RMB)
Cost of drugs	16, 351	19, 731
Cost of laboratory and imaging test	1, 288	1, 352
Cost of adverse event		
Rash	77	236
Diarrhea	11	39
Liver damage	35	15
Cost of hospitalization	265	427
Hypothetical PFS (months)	17.5	13.0
Without medical insurance		
Total costs	315, 472	283, 400
Cost-effectiveness ratio	18, 027	21, 800
ICER per PF-LYS	Referent	1.21
With medical insurance^*^		
Medical insurance ^*^	-262, 500	-195, 000
Total costs	52, 972	88, 400
Cost-effectiveness ratio	3, 027	6, 800
ICER per PF-LYS	Referent	2.25
Guangzhou medical insurance fixed payment procedure was started from February 2010, the patients who enrolled before were excluded from the analysis of cost-effectiveness with medical insurance. ^*^ Guangzhou medical insurance can cover 15, 000 RMB at most for patients per month.		

吉非替尼1疗程治疗药物成本为16, 351元, 厄洛替尼为19, 731元; 影像学及实验室检查成本为1, 288元*vs* 1, 352元; 不良反应费用为265元*vs* 427元。在没有医保报销的情况下, 每位患者相应PFS期限内的治疗总成本平均值, 吉非替尼是315, 472元, 厄洛替尼是283, 400元。CER吉非替尼组为18, 027元/月, 厄洛替尼组为21, 800元/月。ICEA厄洛替尼组为吉非替尼组的1.21倍。

广州市基本医疗保险的最高支付限额每月可以为患者负担15, 000元药费, 大大减低了EGFR-TKI的治疗成本。医保覆盖下的每位患者相应PFS期限内的治疗总成本平均值总计为52, 972元*vs* 88, 400元, 总成本下降明显。CER吉非替尼组为3, 027元/月, 厄洛替尼组为6, 800元/月, 每月治疗成本降至16.8%-31.2%。ICEA厄洛替尼组为吉非替尼组的2.25倍。

## 3 讨论

培美曲塞、吉西他滨、紫杉醇、多西他赛等联合铂类的两药化疗方案自20世纪90年代中期确立其临床地位以来, 至今仍是NSCLC一线治疗的常规方案^[5]^。然而, 近些年来, 靶向药物的异军突起为晚期NSCLC一线治疗带来新的思路。

吉非替尼和厄洛替尼是两个被FDA批准用于晚期NSCLC治疗的EGFR-TKI。近几年多项大型的回顾性或者前瞻性临床研究均显示, 在*EGFR*突变的晚期NSCLC一线或者二线治疗中, 与含铂类化疗药物相比, 两者无论是在治疗效果(ORR, DCR), 还是在生存获益(PFS)上都有一定的优势。IPASS研究的吉非替尼对突变型患者有效率为71.2%, PFS为16.6个月; OPTIMAL研究的厄洛替尼对突变型患者有效率是83%, PFS为13.1个月。EURTAC研究ORR为71%, PFS为9.7个月, WJTOG3405研究ORR为66%, NEJ002以及FIRST SIGNAL等研究结果也相似^[6-10, 15, 16]^。上述大型临床试验结果都说明, 对于*EGFR*突变型的患者的治疗, 吉非替尼或厄洛替尼都优于常规含铂类药物的化疗。

吉非替尼和厄洛替尼拥有基本相同的抗肿瘤机制, 而且在上述众多临床试验中体现出相近的有效率。然而, 对这两种EGFR-TKI的对比研究却十分缺乏。将两者在疗效、生存获益以及治疗成本-效益中进行全面比较, 以确定优效的药物成为研究热点。

本研究纳入了66例在我院接受过吉非替尼或厄洛替尼治疗的广州医保NSCLC患者, 49例(74.2%)服用吉非替尼, 17例(25.8%)服用厄洛替尼。这66例患者均存在*EGFR*基因外显子19或外显子21突变, 且基线状况均衡。

纳入的66例患者均可评估, 进行疗效分析显示吉非替尼和厄洛替尼的RR和DCR相当, RR分别为51.0%和41.2%(*P*=0.578), DCR为91.1%和86.7%(*P*=0.643), 与前文所述临床试验接近; PFS获益在吉非替尼和厄洛替尼中也是比较接近, 分别是17.5个月和13.0个月(*P*=0.459);差异均无统计学意义。不良反应方面, 本研究显示, 使用EGFR-TKI治疗时, 皮疹的发生与疗效的关联无统计学意义(*P*=0.17), 此结论需要进一步研究以证实。吉非替尼组患者的皮疹(62.3%)和腹泻(24.5%)的发生率均稍低于厄洛替尼组(94.1%, 70.6%), 而且3度及以上的重度不良反应的发生率(12.2%, 2.0%)也是低于厄洛替尼组(35.3%, 17.6%)的患者, 其差异有统计学意义(*P* < 0.05)。这些结果与2009年韩国的前瞻性随机Ⅱ期头对头吉非替尼和厄洛替尼二线治疗晚期NSCLC的临床研究所得出的结果很相似^[17]^。2011年台湾一项回顾性研究分析了716例接受过吉非替尼或厄洛替尼治疗的NSCLC患者, 结论也是无论在*EGFR*突变型或野生型患者中, 吉非替尼和厄洛替尼治疗的有效率和生存获益均无差别^[18, 19]^。

从药物治疗成本上考虑, 两种药物也存在一定差别。靶向治疗价格不菲, 许多患者可能因为经济原因无法接受或无法维持靶向治疗。广州是全民医保政策执行较好的城市, 本研究将广州作为范例进行分析, 为全国各地医保政策的制定提供一个参考, 并提供成本-效益分析的方法学模式。从2010年2月1日起广州市的患有NSCLC且*EGFR*突变的医保参保人, 成功申请此项门特项目后可每月获得广州市基本医疗保险的最高支付限额15, 000元, 每月患者的药费从自付近20, 000元降到只需自付几百元, 解决了靶向药物费用昂贵的问题, 让许多患者从中获益。本研究从药物费用、检查费用和相关不良反应的治疗费用计算EGFR-TKI治疗成本, 在患者相应的PFS期限内, 治疗总成本平均值, 吉非替尼是315, 472元, 厄洛替尼是283, 400元; 平均每疗程治疗成本, 吉非替尼18, 027元, 厄洛替尼为21, 800元。广州医保报销后, 总成本下降至52, 972元*vs* 88, 400元, 总成本下降明显; 患者每月的负担分别降至3, 027元*vs* 6, 800元, 成本降至16.8%-31.2%。两种药物之间的对比显示:CER-吉非替尼组为3, 027元/月, 厄洛替尼组为6, 800元/月。ICEA-厄洛替尼组为吉非替尼组的2.25倍。吉非替尼成本-效益比可能会稍优于厄洛替尼。

本研究也存在一些不足之处。首先, 数据来源于单中心、非随机的回顾性研究, 样本量受入组条件限制, 不能像前瞻性临床研究一样达到理想的样本例数和均衡程度, 两组的样本例数偏少, 可能存在一些偏倚, 研究存在一定的局限性。另外, 广州市医保的报销政策是仅基于*EGFR*基因直接测序法, 而不包括PCR法, 这也可能会造成一定的选择偏倚。最后, ICEA分析是在扣除医保负担的费用以后计算的结果, 这种计算方法主要从患者的角度计算有一定的局限性。尽管存在上述不足, 但是这组患者的资料是在非人为干预的情况下获得的一个比较接近真实临床实践的数据, 具有较好的代表性和普遍性。现阶段EGFR-TKI在临床上使用十分广泛, 急需相应的研究作为药物选择的依据, 但是目前这类研究比较缺乏。特别是在目前还缺乏“头对头”临床研究结果, 在大型的前瞻性临床研究结果出来之前, 此文的回顾性分析可以为同道们的临床实践提供一定的参考作用。

综上所述, 本研究回顾性结果分析显示:对于存在*EGFR*外显子19和外显子21敏感突变的晚期NSCLC患者, 接受EGFR-TKI治疗有较好的疗效和生存获益, 而对于TKI的选择, 无论性别、年龄、吸烟与否、突变类型, 吉非替尼和厄洛替尼都具有相当的疗效和生存获益, 而吉非替尼的成本-效益比率可能会稍优于厄洛替尼。这些结果值得进一步前瞻性的大型临床研究去确认。
